# Exploration of potential mechanisms and biomarkers related to ERS-associated RCD in steroid-induced osteonecrosis of the femoral head based on bioinformatics, with experimental validation

**DOI:** 10.3389/fendo.2026.1734283

**Published:** 2026-05-18

**Authors:** Chengbin Yang, Tiansen Zhao, Junhao Xie, Xiangyi Luo, Xiang Tai

**Affiliations:** 1 Department of Trauma Center, First Affiliated Hospital of Kunming Medical University, Kunming, Yunnan, China; 2 Department of Pain Medicine, China Resources Wuhan Iron and Steel General Hospital, Wuhan, Hubei, China; 3 Department of Orthopaedics, The People’s Hospital of Zhenxiong County, Zhaotong, Yunnan, China

**Keywords:** bioinformatics, biomarker, endoplasmic reticulum stress, osteonecrosis of the femoral head, regulated cell death

## Abstract

**Background:**

The pathogenesis of osteonecrosis of the femoral head (ONFH) is associated with endoplasmic reticulum stress (ERS) and regulated cell death (RCD), but reliable linking biomarkers and their mechanisms remain unclear. This study aimed to screen SONFH biomarkers related to ERS/RCD and explore their mechanisms.

**Methods:**

Using datasets from public databases on ONFH, ERS-related genes (ERGs), and RCD-related genes (RRGs), candidate genes were isolated by intersecting the differentially expressed genes (DEGs) with the ERG and RRG sets. We identified biomarkers by integrating a protein-protein interaction (PPI) network, four computational algorithms, and machine learning, with results refined by the Wilcoxon rank-sum test. The diagnostic power of these biomarkers was assessed using receiver operating characteristic (ROC) curve analysis. A subsequent comprehensive investigation included constructing and evaluating a nomogram, along with analyses of pathway enrichment, immune infiltration, drug prediction, and molecular docking, culminating in experimental validation.

**Results:**

Initially, the 100 candidate genes were identified. After multi-step screening, 2 biomarkers (TGFB1, MCL1) were finally determined. Both were significantly upregulated in the ONFH group (p < 0.05), and the area under the ROC curve (AUC) of both was greater than 0.85. The constructed nomogram showed good predictive accuracy (AUC = 0.897, Hosmer-Lemeshow test p = 0.171) and had a net benefit greater than zero, indicating good clinical practicality. These biomarkers were enriched in pathogen-related pathways, correlated with immune cells such as plasmacytoid dendritic cells, and exhibited good molecular docking effects with domperidone (Vina score ≤ -7.7 kcal/mol). RT-qPCR and western blot validation in GC-treated cell models (BMSC, BMEC, THP-1) confirmed significant upregulation of both TGFB1 and MCL1, consistent with the bioinformatics findings.

**Conclusion:**

TGFB1 and MCL1 could serve as potential SONFH biomarkers related to ERS/RCD. The nomogram, as well as the two biomarkers’ regulatory and immune associations, provided a reference for ONFH diagnosis. Domperidone shows potential as a candidate drug for the targeted therapy of ONFH, although its clinical application remains pending *in-vivo* efficacy and clinical cohort validation.

## 1 Introduction

Osteonecrosis of the femoral head (ONFH) is a debilitating orthopedic disease in young and middle-aged adults, caused by disrupted blood supply that leads to osteocyte death and structural collapse, ultimately resulting in joint dysfunction, a high risk of disability, and a heavy burden on patients’ quality of life and healthcare systems (1, 2). Although risk factors such as corticosteroid use, trauma, and alcohol abuse have been clearly linked to its onset (3, 4), the molecular mechanisms driving disease progression remain poorly understood, particularly the role of cell death, which has yet to be systematically explored. Endoplasmic reticulum stress (ERS) is increasingly recognized for its crucial role in protein homeostasis and stress regulation, with its dysregulation linked to bone metabolic imbalance. Meanwhile, regulated cell death (RCD) processes, such as apoptosis and pyroptosis, are established as key contributors to bone homeostasis (5–7).

Through its three major transmembrane receptors on the ER membrane, ERS has been shown to trigger various forms of cell death, such as autophagy, apoptosis, ferroptosis, and pyroptosis (8). In glucocorticoid-induced osteonecrosis of the femoral head, ERS is closely associated with osteoblast apoptosis through the PERK/CHOP pathway, where CHOP can inhibit Bcl-2 expression and increase activated caspase-3 levels, leading to cell death (9). Studies have found that eIF2α signaling regulates ischemic osteonecrosis through endoplasmic reticulum stress, and iron overload can induce osteoblast apoptosis by triggering ERS-mediated mitochondrial dysfunction and the p-eIF2α/ATF4/CHOP pathway (10). These studies collectively indicate that there is a close molecular interaction between ERS and RCD, forming an important signaling axis that regulates cell fate. In addition, in osteoporosis and osteoarthritis, ERS and various forms of RCD are deeply involved in disease progression by regulating osteogenesis, osteoclast activity, and inflammatory responses (6, 11). These findings suggest that the ‘ERS-RCD axis’ may be a core component in the pathogenesis of ONFH. However, systematic research on the ERS-RCD interaction in the field of ONFH remains insufficient, and its underlying molecular network mechanisms have not been fully elucidated. More importantly, current studies mostly focus on individual genes or pathways (such as PI3K/AKT-FOXO1) (12). Targeted therapeutic approaches for ONFH are also extremely limited, and currently, there have been almost no attempts at drug development based on the ‘ERS-RCD axis’. Targeted therapeutic approaches for ONFH are also extremely limited, and currently, there have been almost no attempts at drug development based on the ‘ERS-RCD axis’.

By combining bioinformatics analysis of GEO transcriptomic data with experimental validation, this study identified ERS- and RCD-related biomarkers in SONFH. The analytical workflow encompassed nomogram construction, functional enrichment, immune infiltration, and drug prediction. These results help clarify the “ERS-RCD axis” in ONFH pathogenesis, translate computational findings into experimental evidence, and suggest potential new avenues for clinical treatment.

## 2 Materials and methods

### 2.1 Data collection

Data for this study were sourced from the Gene Expression Omnibus (GEO) database (https://www.ncbi.nlm.nih.gov/geo/), comprising two ONFH datasets: the training set GSE123568 and the test set GSE74089. The training set (GSE123568) comprised 30 peripheral serum samples from patients with steroid-induced ONFH (SONFH group) and 10 from those with non-steroid-induced ONFH (non-SONFH group, serving as the subtype control). This setup was primarily employed to identify biomarkers and mechanisms specifically associated with the progression of steroid-induced pathology. This dataset was primarily employed for differential expression analysis and subsequent investigations. The sequencing platform was GPL15207. Similarly, the 4 cartilage tissue samples from ONFH patients (ONFH group) and 4 cartilage tissue samples from healthy controls (control group) in the test set were included for gene expression validation. The sequencing platform was GPL13497.

The GeneCards^®^ database (https://www.genecards.org/) was queried using the keyword “endoplasmic reticulum stress” with a relevance score threshold set at >5, yielding a total of 1,520 ERGs. (**Supplementary Table 1**). Additionally, genes related to 13 different types of regulated cell death (RCD) were collected by integrating 3 databases (Gene Set Enrichment Analysis (GSEA) gene sets, Kyoto Encyclopedia of Genes and Genomes (KEGG, https://www.kegg.jp/), and Ferroptosis Database (FerrDb, http://www.zhounan.org/ferrdb/current/) and relevant literature (13). After merging and deduplication, 1,282 RCD-related genes (RRGs) were acquired (**Supplementary Table 2**). The strategic inclusion of disparate biological sources—peripheral serum for the training set (GSE123568) and cartilage tissue for the validation set (GSE74089)—was intended to identify robust biomarkers that are consistently dysregulated at both the systemic and local levels. Peripheral serum, as a non-invasive and clinically accessible sample, is ideal for identifying biomarkers suitable for early screening and clinical translation. Conversely, cartilage tissue, being the direct site of the lesion, provides a localized reflection of the pathological microenvironment, including ERS and RCD processes. This cross-tissue approach ensures that the identified markers possess both diagnostic accessibility and direct pathological relevance to ONFH.

### 2.2 Identification and analysis of candidate genes

Using the “limma” package (v 3.56.2) (14), we first screened for DEGs in the training set (ONFH vs. control; criteria: |log2FC| > 0.5, p < 0.05). Subsequently, a volcano plot was generated with “ggplot2” (v 3.5.2) (15) to visualize these DEGs, with labels for the top 10 genes based on |log2FC|. A heatmap focusing on these top genes was then produced using the “ComplexHeatmap” package (v 2.16.0) (16). To identify candidate genes, we performed an intersection analysis of the DEGs with ERGs and RRGs utilizing the “ggVennDiagram” package (v 1.5.2) (17).

To elucidate the biological functions and signaling pathways associated with the candidate genes, functional enrichment was assessed via Gene Ontology (GO) and Kyoto Encyclopedia of Genes and Genomes (KEGG) analyses using the “clusterProfiler” package (v 4.2.2) (18), applying a p.adjust cutoff of < 0.05. A subset of the significantly enriched terms was visualized by sorting them in descending order based on the gene count. We performed a Disease Ontology (DO) enrichment analysis (p.adjust < 0.05) via The Disease Ontology Knowledgebase (DO-KB)(https://disease-ontology.org/) to clarify the disease associations and mechanisms of the candidate genes. This provided functional clues for analyzing their role in ONFH pathogenesis, and the top 10 results ranked by GeneRatio are shown.

The protein-protein interaction (PPI) analysis involved three sequential steps. First, candidate genes were submitted to the Search Tool for the Retrieval of Interacting Genes/Proteins (STRING) database (http://string-db.org) to generate a PPI network (interaction score ≥0.15). Next, network topology was characterized using Cytoscape’s Cytohubba (v 3.10.2) (19) plugin, which calculated node connectivity through four distinct algorithms: Degree, Density of Maximum Neighborhood Component (DMNC), Maximum Clique Centrality (MCC), and Maximum Neighborhood Component (MNC). Finally, core genes were derived from the intersection of the top 30 genes identified by each algorithm.

### 2.3 Acquisition of biomarkers and exploration of their ability to distinguish samples

Core gene screening was conducted via least absolute shrinkage and selection operator (LASSO) regression analysis with 5-fold cross-validation on the training set, implemented using the “glmnet” package (v 4.1.4) (20). When the lambda parameter took the minimum (min) value, the model exhibited the minimum cross-validation error, and at this time, the first set of feature genes was successfully selected through L1 norm penalty. In the training set, feature selection was performed using multiple approaches. An initial set of feature genes was identified via the Boruta algorithm, retaining only those marked as “confirmed”. Additionally, support vector machine-recursive feature elimination (SVM-RFE) analysis with 5-fold cross-validation was conducted using the “caret” (v 6.0.94) (21) and “e1071” (v 1.7.13) (22) packages, yielding a third set of feature genes. Yet in the training set, the “xgboost” package (v 2.1.1.1) (23) was used to implement the eXtreme Gradient Boosting (XGBoost) algorithm for feature selection, and the fourth set of feature genes with gain > 0.1 was screened. To identify the overlapping feature genes, we performed an intersection analysis of the aforementioned four gene sets using the “VennDiagram” package (v 1.7.3) (24).

Candidate biomarkers for ONFH, ERS, and RCD were screened by assessing the expression of the intersecting feature genes. This was done by applying the Wilcoxon rank-sum test (p < 0.05) to compare the ONFH and control groups in both the training and test sets. A gene was ultimately designated as a biomarker only if it showed significant differential expression and a consistent trend of change across both datasets.

The discriminatory power of the biomarkers for different sample types was assessed by conducting receiver operating characteristic (ROC) curve analysis and calculating the area under the curve (AUC) in the training set, using the “pROC” package (v 1.18.5) (24). When AUC > 0.7, the biomarker was considered to have good performance. To justify the implementation of the ensemble approach, the predictive performance of the feature sets identified by each of the four individual machine learning algorithms was assessed via ROC analysis. These values were then compared with the performance of the final intersected gene set to evaluate whether the dimensionality reduction achieved through the intersection strategy maintained robust diagnostic accuracy.

### 2.4 Multidimensional analysis of biomarkers

For the assessment of the biomarkers’ predictive power for ONFH, a nomogram was constructed in the training set using the “rms” package (25). The nomogram consisted of two parts: “Points” (scores of individual biomarkers) and “Total Points” (total scores of all biomarkers). Multiple validation methods were employed to assess the nomogram. Calibration was evaluated using the “rms” package (v 6.8.1), with adequate fit defined by a non-significant Hosmer-Lemeshow test (p > 0.05). Discrimination was assessed by plotting an ROC curve and calculating the AUC with the “pROC” package (v 1.18.5), where an AUC between 0.7–1 was considered indicative of good predictive performance. Additionally, decision curve analysis performed with the “rmda” package (v 1.6) (26) quantified the clinical utility of the nomogram.

The functional profiling of biomarkers proceeded through three complementary analytical phases. First, a genome-wide Spearman correlation analysis was conducted in the training set using the “psych” package (v 2.4.6.26) (27), producing a ranked list of associated genes. Second, pathway-level enrichment was assessed concurrently through GSEA implemented with the “clusterProfiler” package (v 4.2.2) and the c2.cp.kegg.v7.4.symbols.gmt gene set from MSigDB (http://www.gsea-msigdb.org/gsea/msigdb/), from which the top five pathways meeting |NES| > 1, FDR < 0.25, and p < 0.05 were selected. Third, to investigate pathway heterogeneity, ONFH samples were dichotomized by median biomarker expression, and GSVA was executed using the “GSVA” package (v 1.53.28) (28) with the h.all.v7.0.symbols.gmt gene set, yielding results that were filtered (|t| > 2, p < 0.05) for final presentation.

Given established evidence linking immune infiltration to ONFH pathogenesis through regulation of inflammation, angiogenesis, and bone metabolism (29), we characterized the immune microenvironment in ONFH. Our analysis of the training set compared the ONFH and control groups using the ssGSEA algorithm (“GSVA” package v 1.53.28) to quantify 28 immune cell populations. Immune cells showing significant abundance differences by Wilcoxon rank-sum test (p < 0.05) were designated as differentially immune cells (DICs). Correlation analyses between DICs, and between biomarkers and DICs, were further conducted with the “psych” package (v 2.4.6.26), applying thresholds of |cor| > 0.3 and p < 0.05.

We characterized ONFH heterogeneity and associated immune patterns through a two-stage analysis. First, biomarker-based consensus clustering was conducted on ONFH samples in the training set using the “ConsensusClusterPlus” package (v 1.71.0) (30) implementing the K-means algorithm, with cluster numbers ranging from 2–10 to identify the optimal stratification. Visualization packages (“ggplot2” v 3.5.2) produced consensus clustering results, CDF, delta area, and tracking plots. Second, to delineate immune characteristics across subtypes, we quantified infiltration levels of 28 immune cell types using Z-score normalization and compared these scores between subtypes with statistical significance (p < 0.05).

Biomarker localization analysis comprised two complementary components: chromosomal mapping executed with the “RCircos” package (v 1.2.2) (31), and subcellular localization prediction carried out through the CELLO system (https://cello.life.nctu.edu.tw/).

To explore the molecular regulatory mechanisms of biomarkers, transcription factors (TFs) that might interact with biomarkers were predicted using the NetworkAnalyst tool (https://networkanalyst.ca/) combined with the JASPAR database (https://jaspar.elixir.no/), and microRNAs (miRNAs) that could interact with biomarkers were predicted via miRDB (https://mirdb.org/). Meanwhile, long non-coding RNAs (lncRNAs) related to biomarkers were predicted by combining the Encyclopedia of RNA Interactomes (ENCORI/starBase, http://starbase.sysu.edu.cn/). Our analysis extended to constructing TF-mRNA and lncRNA-miRNA-mRNA networks, complemented by an investigation into biomarker interactions and functional associations via the GeneMANIA database (https://genemania.org).

Finally, to explore potential drugs for the treatment of ONFH, potential drugs that interact with biomarkers were predicted using the Drug Signatures Database (DSigDB, https://dsigdb.tanlab.org/DSigDBv1.0/). Drugs with multi-target regulatory potential were screened by obtaining the intersection of the two sets of drugs, and highly toxic substances among them were excluded. Drugs that interacted more closely with biomarkers were screened based on “Interaction.Count”, and the top 20 drugs were further selected using “Combined.Score” and “p” to construct a biomarker-drug association network. On this basis, the top 1 drug was selected from these 20 drugs again using “Combined.Score” and “p” for molecular docking with biomarkers. The protein structures of biomarkers were obtained from the Uniprot database (https://www.uniprot.org/), and the three-dimensional structures of the top 1 drug were downloaded from the PubChem database (https://pubchem.ncbi.nlm.nih.gov/). These two structures were imported into the online tool CB-DOCK2 (https://cadd.labshare.cn/cb-dock2/index.php) for docking. Generally, a docking score (Vina score) < -5.0 kcal/mol was considered to indicate good binding activity.

### 2.5 Reverse transcription-quantitative polymerase chain reaction

The cell lines used in this study were human brain microvascular endothelial cells (BMEC), human monocytic leukemia cells (THP-1), and bone marrow mesenchymal stem cells (BMSC). Cells were cultured as follows: BMEC – 89% DMEM/F12, 10% FBS (Gibco, Cat. No. 10099-141), 1% penicillin/streptomycin (Melen Bio, Cat. No. MA0110); THP-1 – RPMI 1640 medium (vivecall, Cat. No. C3113-0500) with 10% FBS and 1% penicillin/streptomycin; hBMSCs – DMEM low-glucose medium (Solarbio, Cat. No. 31600) with 10% FBS and 1% penicillin/streptomycin. All three types of cells were incubated in a humidified constant-temperature incubator at 37°C with 5% CO2. Briefly, the above three cell lines were first revived and cultured. When the confluency of these three cell lines reached 90%, they were passaged at a ratio of 1:2. After passage, part of the cells was continuously cultured until cryopreservation, while the other part was used for the following operations. THP-1 cells were pretreated with 100 ng/mL PMA (MCE, Cat. No. HY-18739) for 24 hours; thereafter, the medium was refreshed for continued culture. When the confluency of BMEC, THP-1, and BMSC reached 80%, they were treated with 100 nM dexamethasone (glucocorticoid (GC); APExBIO, Cat. No. K1018-5ml) for 48 hours. After 48 hours of GC treatment, the cells (BMEC+GC, THP-1+GC, BMSC+GC) were collected. For the collected cells (BMEC+GC, THP-1+GC, BMSC+GC, and BMSC; three replicates for each cell line), the old medium was discarded first, followed by washing with phosphate-buffered saline (PBS; Solarbio, Cat. No. BL302A) which was then discarded. Following RNA extraction (Trizol reagent; Vazyme, China) and quantification (NanoPhotometer N50), reverse transcription was carried out using the specified kit (Younggen, China). RT-qPCR analyses were performed in triplicate on a CFX system (Bio-Rad, USA). The 2-ΔΔCt method normalized to GAPDH was applied to calculate relative biomarker expression. Data analysis utilized GraphPad Prism 10 (32), with intergroup differences evaluated by Student’s t-test. Primer sequences used in this study are documented in **Supplementary Table 1**.

### 2.6 Western blot

Total protein was extracted using RIPA buffer, and the protein concentration was quantified. After SDS-PAGE and membrane transfer, the membranes were incubated with primary antibodies against TGFB1, MCL1, and GAPDH. Signals were detected via enhanced chemiluminescence.

### 2.7 Statistical analysis

All analyses were carried out in R (v 4.3.3), considering p < 0.05 as statistically significant. The Wilcoxon rank-sum test was used for cohort comparisons, and the Student’s t-test was applied for RT-qPCR data analysis.

## 3 Results

### 3.1 Acquisition and analysis of candidate genes related to ERS and RCD in ONFH

**Figure 1:**
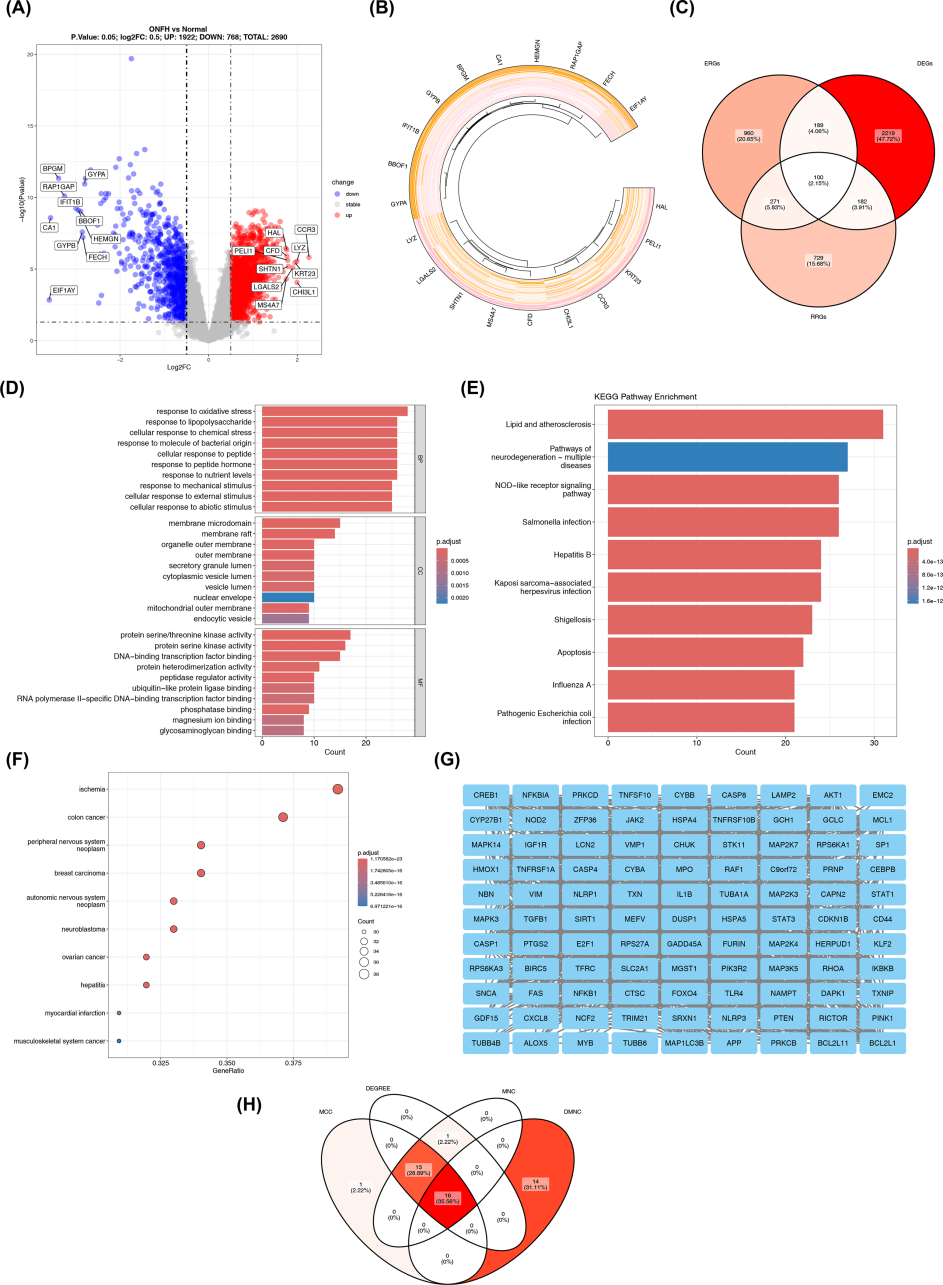
Acquisition and analysis of candidate genes related to ERS and RCD in ONFH. **(A)** Volcano plot of DEGs; **(B)** Heatmap of the labeled genes; **(C)** Acquisition of candidate genes; **(D-E)** GO/KEGG analysis; **(F)** DO enrichment analysis; **(G)** Construction of PPI network; **(H)** Acquisition of core genes.

A total of 2,690 DEGs (|log_2_FC| > 0.5, p < 0.05) were screened from the training set. Among them, compared with the control group, there were 1,922 upregulated genes and 768 downregulated genes in the ONFH group (**Supplementary Table 3**). The volcano plot of DEGs and the heatmap of labeled genes were shown in **Figures 1A, B**, respectively. Subsequently, the 100 candidate genes were further obtained through intersection analysis (**Figure 1C**; **Supplementary Table 4**). GO enrichment analysis for the candidate genes identified a total of 1,595 functional terms. The results were categorized into 1,463 terms for Biological Process (BP), 56 for Cellular Component (CC), and 76 for Molecular Function (MF). These terms implicated a spectrum of critical functions, including responses to oxidative stress, lipopolysaccharide, and chemical stress; cellular components such as membrane microdomains, membrane rafts, and the organelle outer membrane; and molecular functions like protein serine/threonine kinase activity, protein serine kinase activity, and DNA-binding transcription factor binding (**Supplementary Table 5**; **Figure 1D**); KEGG enrichment analysis showed that it covered 158 pathways, including pathways of neurodegeneration-multiple diseases, nucleotide-binding oligomerization domain (NOD)-like receptor signaling pathway, and apoptosis (**Supplementary Table 6**; **Figure 1E**) (p.adjust < 0.05). Candidate genes were significantly linked to 619 human diseases in the DO enrichment analysis, exemplified by ischemia, colon cancer, and peripheral nervous system neoplasm (**Supplementary Table 7**; **Figure 1F**) (p.adjust < 0.05).

And the PPI network constructed based on candidate genes (**Figure 1G**) showed that there were 1,283 interaction relationships (after removing 1 isolated target), and multiple candidate genes interacted with each other to form a relatively tight “interaction cluster”. By intersecting the top 30 genes identified by the four algorithms, we obtained 16 core genes (**Figure 1H**; **Supplementary Tables 8**-**12**).

### 3.2 Acquisition of biomarkers related to ERS and RCD in ONFH and evaluation of their ability to distinguish samples

**Figure 2:**
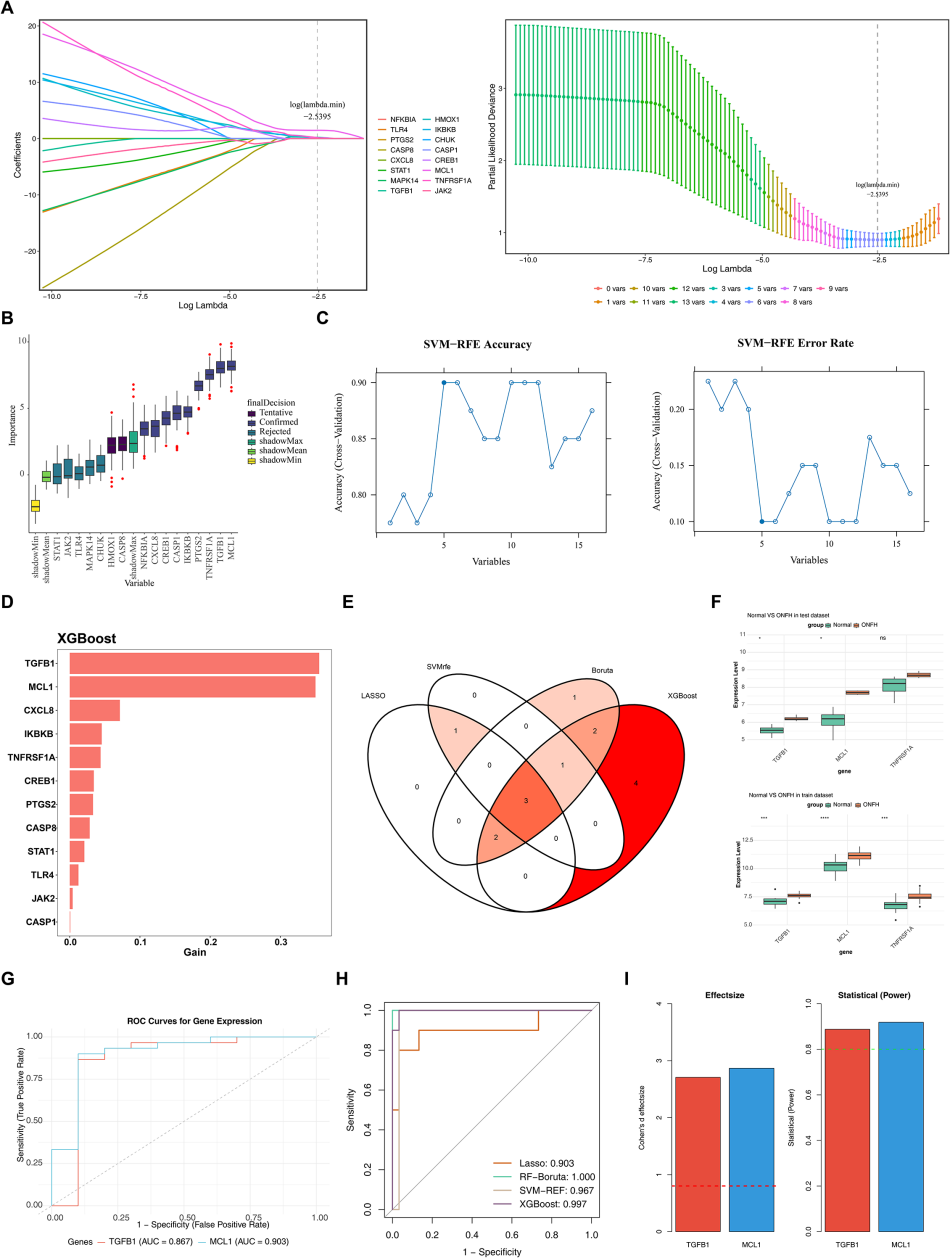
Identification of biomarkers and evaluation of their ability to distinguish samples. **(A)** LASSO regression analysis; **(B)** Feature selection analysis; **(C)** SVM-RFE analysis; **(D)** XGBoost algorithm analysis; **(E)** Acquisition of core genes; **(F)** Expression validation of core genes. *p < 0.05, **p < 0.01, ***p < 0.001, ****p < 0.0001, and ns indicates no significance. **(G)** ROC curve analysis of biomarkers. **(H)** ROC curves of the feature sets identified by the four individual machine learning algorithms in the training set. The AUC values are explicitly labeled, providing a baseline for assessing the diagnostic performance gains of the intersection-based ensemble approach. **(I)** *Post-hoc* power analysis of key genes in the validation set (GSE74089). The left panel illustrates the Cohen’s d effect sizes for TGFB1 and MCL1, with the red dashed line indicating the conventional threshold for a large effect (d = 0.8). Both genes exhibit exceptionally large effect sizes. The right panel displays the corresponding *post-hoc* statistical power, where the green dashed line represents the standard threshold for statistical sufficiency (Power=0.8).

Machine learning of four algorithms (LASSO, Boruta, SVM-RFE, XGBoost) was carried out for core genes, and four groups of feature genes were successfully screened, as follows: As shown in **Figure 2A**, when log (lambda.min) = -2.5395, the model had the smallest cross-validation error, and 6 feature genes (TGFB1, HMOX1, IKBKB, CREB1, MCL1, TNFRSF1A) were screened (the first group of feature genes). As shown in **Figure 2B**, there were 9 feature genes with the status of “confirmed”, namely NFKBIA, PTGS2, CXCL8, TGFB1, IKBKB, CASP1, CREB1, MCL1, and TNFRSF1A (the second group of feature genes). As shown in **Figure 2C**, when the number of feature genes was 5, the prediction error of the model was the lowest and the performance was the best, and the included genes were MCL1, TNFRSF1A, TGFB1, HMOX1, and PTGS2 (the third group of feature genes). As shown in **Figure 2D**, there were 12 genes with gain > 0.1, namely TGFB1, MCL1, CXCL8, IKBKB, TNFRSF1A, CREB1, PTGS2, CASP8, STAT1, TLR4, JAK2, and CASP1 (the fourth group of feature genes). After taking the intersection of the above four groups of feature genes, 3 intersection feature genes were finally obtained, namely TGFB1, MCL1, and TNFRSF1A (**Figure 2E**).

The Wilcoxon rank-sum test revealed consistent and significant expression differences for TGFB1 and MCL1. In both the training and test sets, these genes were significantly upregulated in the ONFH group compared to the control group (p < 0.05; **Figure 2F**). The final biomarkers, TGFB1 and MCL1, demonstrated outstanding diagnostic efficacy in the training set. Their AUC values (0.867 and 0.903, respectively) substantially surpassed the 0.700 cutoff, affirming their capability to effectively differentiate ONFH samples from controls (**Figure 2G**). To evaluate the performance gains of the ensemble approach, ROC analysis was conducted for each feature set identified by the four individual algorithms in the training set (GSE123568). The Area Under the Curve (AUC) values for LASSO, RF-Boruta, SVM-RFE, and XGBoost were 0.903, 1.000, 0.967, and 0.997, respectively (**Figure 2H**). To evaluate the statistical reliability of the validation results, a *post-hoc* power analysis was performed for TGFB1 and MCL1 in the test set (GSE74089, n = 4 per group). The Cohen’s d effect sizes for TGFB1 and MCL1 were [2.71] and [2.87], respectively, both exceeding the threshold for a large effect (d > 0.8). Consequently, the calculated statistical power values were [0.88] for TGFB1 and [0.92] for MCL1 (**Figure 2I**), both of which surpassed the conventional sufficiency threshold of 0.8. These results indicate that the observed differential expressions are statistically robust despite the limited sample size.

### 3.3 Biomarkers: nomogram construction and evaluation, pathway enrichment, and immune infiltration

**Figure 3:**
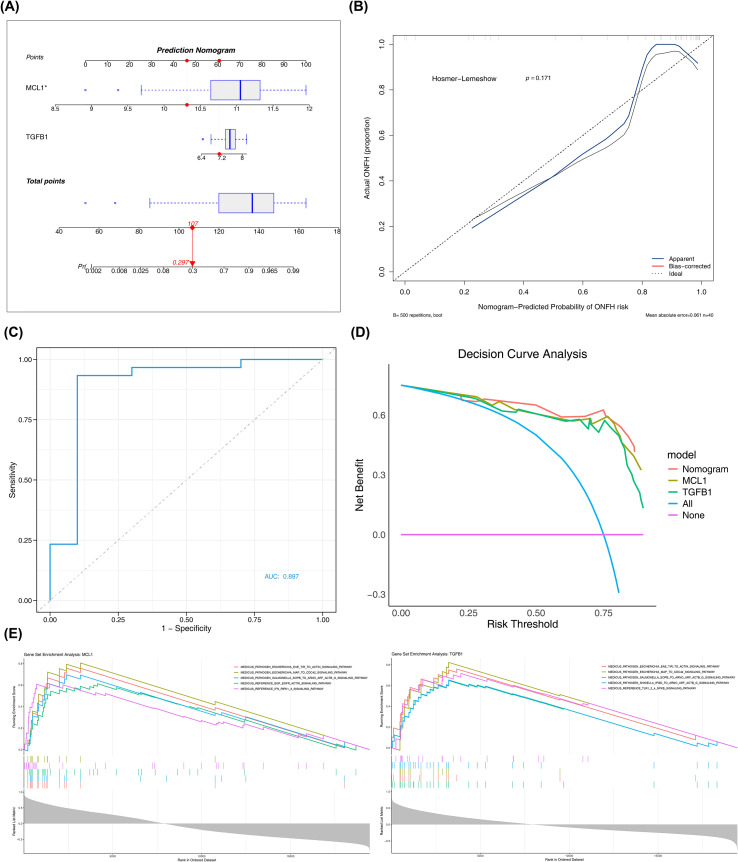
Construction of the nomogram and GSEA enrichment analysis of biomarkers. **(A)** Construction of nomogram. The points represent the score of each variable, with the total points for the two biomarkers, TGFB1 and MCL1, calculated and mapped to the linear score and outcome probability; **(B)** Calibration curve of the nomogram; **(C)** ROC curve of the nomogram; **(D)** Decision curve analysis of the nomogram; **(E)** GSEA enrichment analysis of biomarkers. (Left) MCL1; (Right) TGFB1. Different colors represent different pathways.

The nomogram was constructed for the two selected biomarkers, TGFB1 and MCL1 (**Figure 3A**). According to the nomogram model, by calculating the total score, the probability of an individual suffering from ONFH could be inferred. The higher the total score, the greater the risk of illness. On this basis, the calibration curve was further drawn (**Figure 3B**). As a preliminary exploration of the clinical diagnostic potential of the identified biomarkers, a nomogram was constructed based on TGFB1 and MCL1. Although the model demonstrated high discriminative power with an AUC of 0.897, these statistical results are intended to provide a theoretical framework for future clinical applications rather than serving as a finalized diagnostic tool, particularly given the current sample size constraints (**Figure 3C**), while decision curve analysis (**Figure 3D**) demonstrated clinical applicability with net benefit values consistently above zero.This indicated that the nomogram could effectively predict the risk of illness in individuals on the basis of integrating TGFB1 and MCL1, showing good application prospects and practical value.

**Figure 4:**
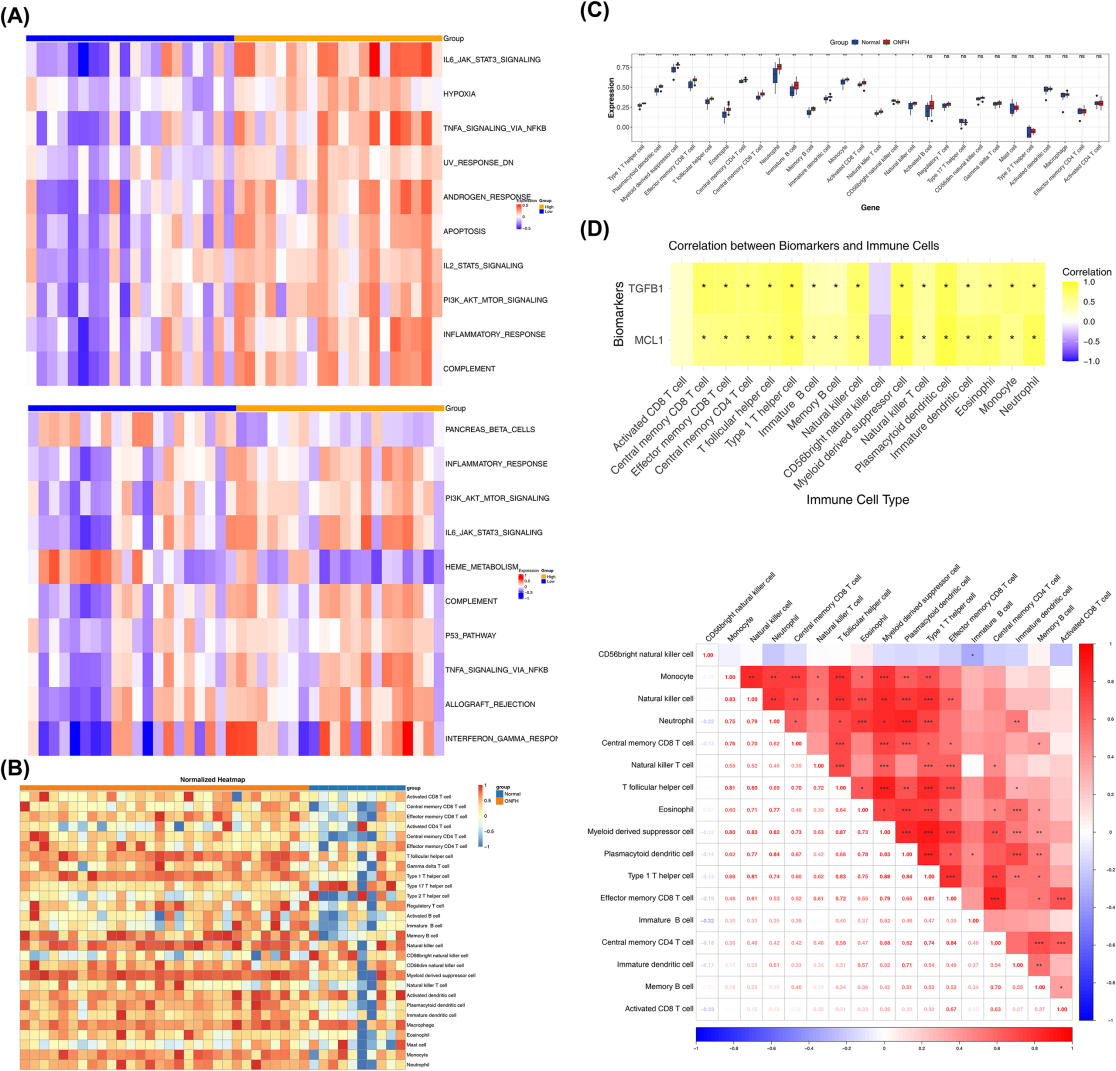
GSVA pathway enrichment analysis and immune infiltration analysis of biomarkers. **(A)** GSEA enrichment analysis of biomarkers. (Top) MCL1; (Bottom) TGFB1; **(B)** Heatmap of immune cell scores in the normal group and ONFH group for 28 immune cells; **(C)** Boxplot of differential immune cells. *p < 0.05, **p < 0.01, ***p < 0.001, and ns indicates no significance. (**D**). Correlation analysis. (Top) Heatmap of correlations between differential immune cells, and (Bottom) heatmap of correlations between biomarkers and differential immune cells. *p < 0.05, **p < 0.01, ***p < 0.001.

Then, through GSEA, it was found that MCL1 was enriched in 146 pathways, including medicus pathogen *Escherichia* EAE TIR to ACTIN signaling pathway, medicus pathogen *Escherichia* map to CDC42 signaling pathway, medicus pathogen *Salmonella* Sopb to Arno ARF ACTB G signaling pathway, medicus reference EGF EGFR ACTIN signaling pathway, and medicus reference IFN RIPK1/3 signaling pathway, etc.; TGFB1 was enriched in 130 pathways, including medicus pathogen *Escherichia* EAE TIR to ACTIN signaling pathway, medicus pathogen *Escherichia* map to CDC42 signaling pathway, medicus pathogen *Salmonella* Sopb to Arno ARF ACTB G signaling pathway, medicus pathogen *Shigella* Ipgd to Arno ARF ACTB G signaling pathway, and medicus reference TLR1/2/4 NF-κB_signaling pathway, etc. (**Figure 3E**; **Supplementary Tables 13**, **14**). This suggested that TGFB1 and MCL1 might be involved in related biological processes by regulating pathogen-related and specific reference signaling pathways. At the same time, GSVA found that in the high and low expression groups of MCL1, 21 pathways were activated and 3 pathways were inhibited, for example, IL-6/JAK2/STAT3 signaling, hypoxia, TNF-α signaling via NF-κB, etc. were significantly activated (t > 2, p < 0.05), and spermatogenesis, KRAS signaling DN, heme metabolism, etc. were significantly inhibited (t < -2, p < 0.05); while in the high and low expression groups of TGFB1, 15 pathways were activated and 2 pathways were inhibited, for example, inflammatory response, PI3K-AKT-mTOR signaling, IL-6/JAK2/STAT3 signaling, etc. were significantly activated (t > 2, p < 0.05), and pancreas beta cells, heme metabolism were significantly inhibited (t < -2, p < 0.05) (**Figure 4A**; **Supplementary Tables 15**, **16**). These enrichment results suggest that systemic immune activation and pathogen-associated molecular patterns (PAMPs) signaling may serve as upstream drivers or systemic reflections of localized bone injury. The predominance of immune-related pathways highlights the involvement of the ‘bone-immune axis’ in the pathogenesis of steroid-induced ONFH, where systemic inflammatory mediators potentially modulate the microenvironment of the femoral head, predisposing it to necrotic changes. The above results showed that TGFB1 and MCL1 might be involved in related biological processes or disease occurrence and development by regulating the activation and inhibition of common pathways and their respective specific pathways.

Next, immune infiltration analysis was carried out. **Figure 4B, C** illustrate the differential immune landscape: Myeloid-derived suppressor cells were enriched in the ONFH group, while CD56bright natural killer cells were more abundant in controls. A total of 17 immune cell subsets, including Type 1 T helper cells and Plasmacytoid dendritic cells, were defined as DICs with statistical significance (p < 0.05). At the same time, as shown in **Figure 4D** and **Supplementary Table 17**, there was a significant positive correlation between most DICs, among which Type 1 T helper cells and Myeloid derived suppressor cells had the strongest positive correlation (cor = 0.88, p < 0.001); only Immature B cells and CD56bright natural killer cells had a negative correlation (cor = -0.32, p < 0.05); MCL1 and TGFB1 exhibited the strongest positive correlations with Plasmacytoid dendritic cells (cor = 0.87, 0.81, p < 0.05) among their significant positive associations with most DICs. This correlation pattern suggests these biomarkers potentially influence ONFH progression through regulating immune cell activity.

### 3.4 Other dimensional analyses of biomarkers

**Figure 5:**
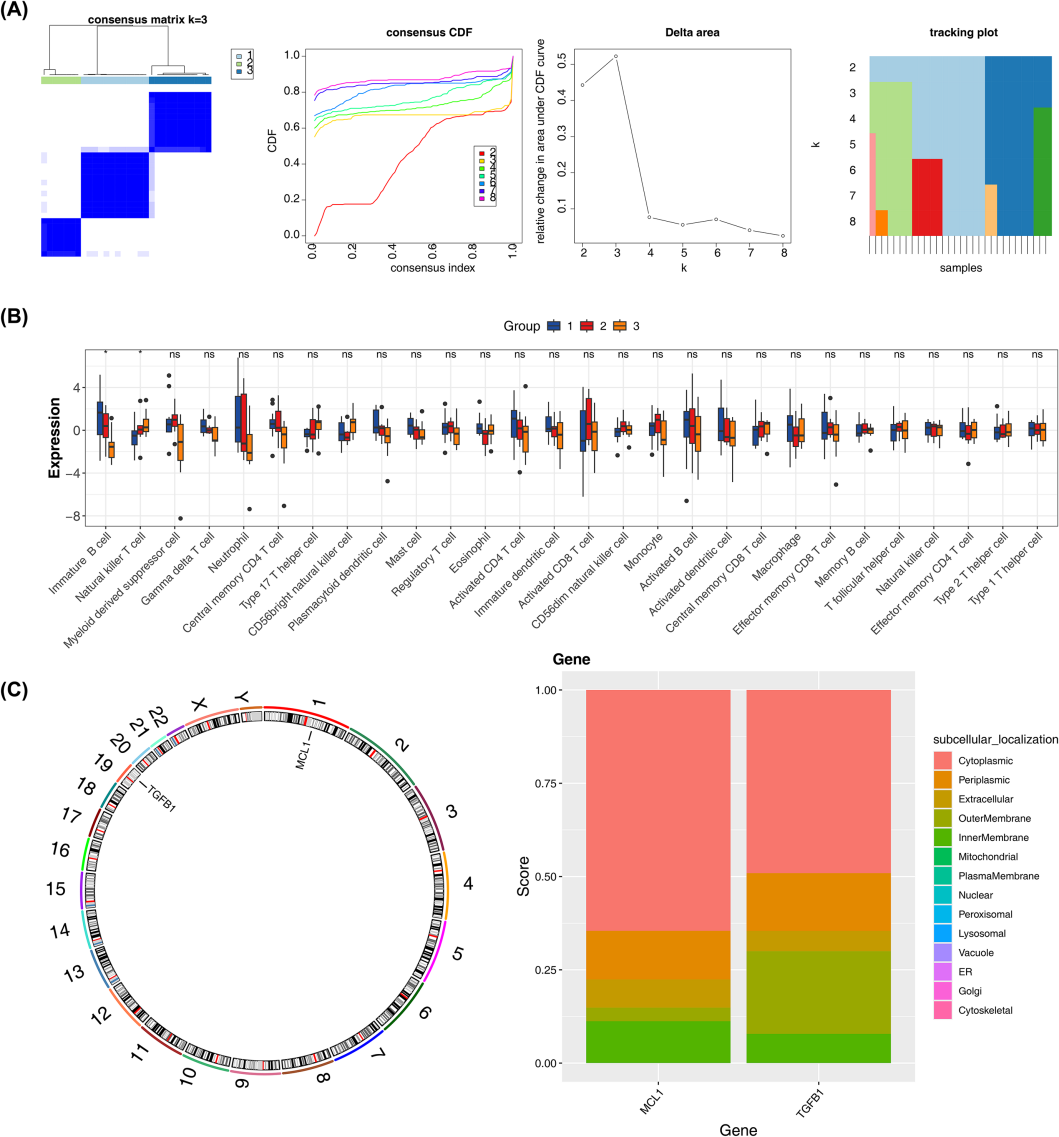
Multidimensional analysis of biomarkers. **(A)** Identification results of the three ONFH subtypes; **(B)** Differences in immune cell infiltration among different subtypes. *p < 0.05 and ns indicates no significance; **(C)** (Left) Chromosomal localization and (Right) subcellular localization distribution of biomarkers. Different colors represent different locations within the cell.

The optimal number of clusters was determined to be 3 via consensus clustering analysis, meaning the ONFH patients could be divided into 3 subtypes (**Figure 5A**). Additionally, as shown in **Figure 5B**, among the 28 types of immune cells, only the infiltration levels of Immature B cells and Natural killer T cells showed significant differences among the 3 ONFH subtypes (p < 0.05), while the infiltration levels of the remaining 26 types of immune cells exhibited no statistical differences among the subtypes.

Subsequently, the chromosome/subcellular localization of biomarkers (**Figure 5C**) showed that MCL1 was located on chromosome 1 and mainly expressed in the cytoplasm, while TGFB1 was located on chromosome 19 and also expressed in the cytoplasm.

**Figure 6:**
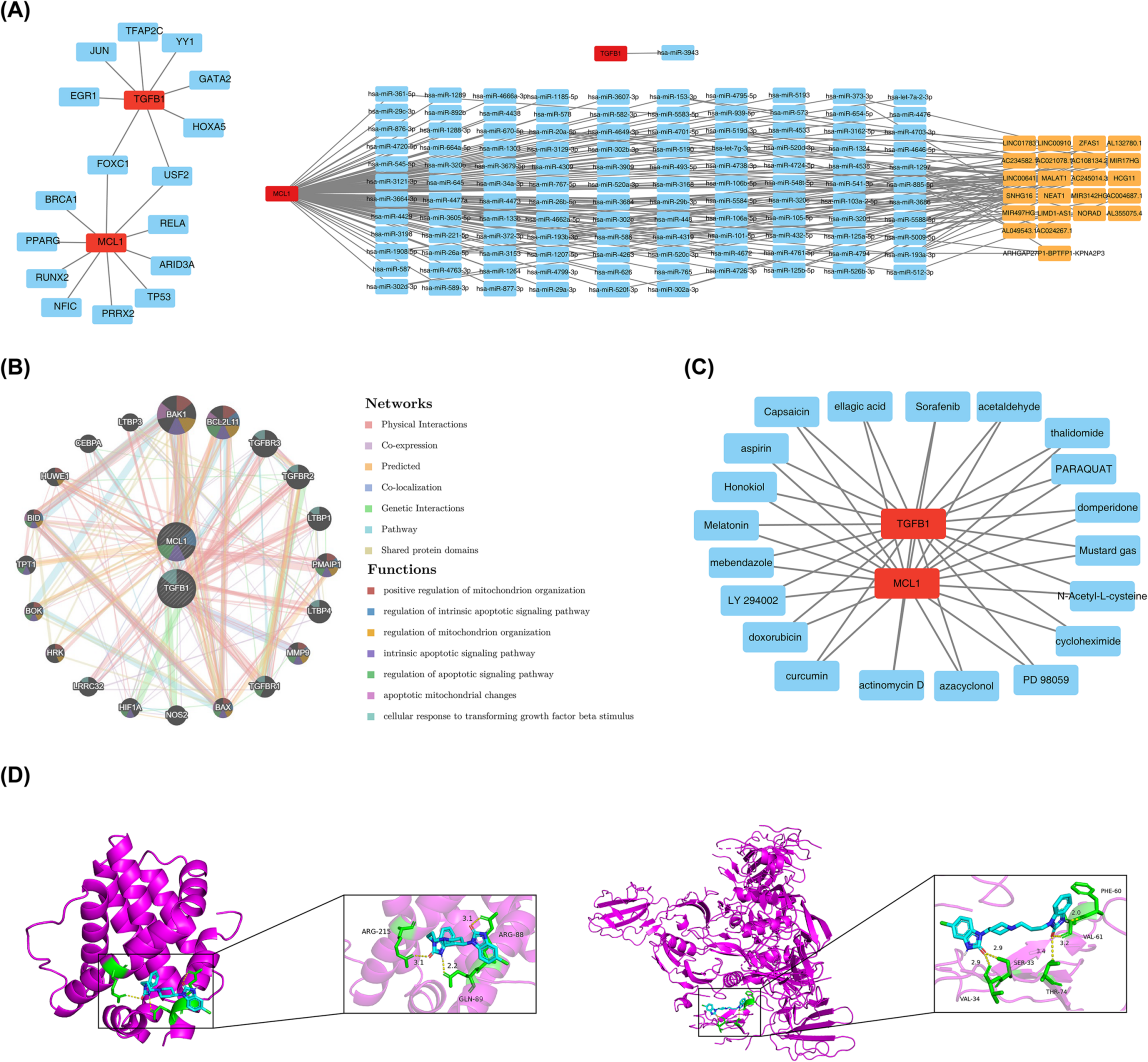
Molecular regulatory network, drug prediction, and molecular docking. **(A)** Analysis of molecular regulation. (Left) TF-mRNA network. Red represents biomarkers, and blue represents associated transcription factors; (Right) lncRNA-miRNA-mRNA network. Red represents biomarkers, blue represents miRNAs, and yellow represents lncRNAs; **(B)** GeneMANIA network; **(C)** Drug prediction. Red represents biomarkers, and blue represents predicted drugs. **(D)** Three-dimensional (3D) representations of the molecular docking models between domperidone and the target proteins (MCL1 and TGFB1). The 3D binding diagrams illustrate the spatial orientation of the ligand within the binding pockets. Interacting amino acid residues and the ligand are displayed as stick models, with key hydrogen bonds (H-bonds) and their corresponding distances in Angstroms (Å) explicitly labeled to demonstrate the stability of the binding.

Moreover, in-depth analysis was carried out on the regulatory mechanism of biomarkers. The results showed that TGFB1 could be regulated by 8 TFs and 1 miRNA; MCL1 was regulated by 10 TFs, 116 miRNAs, and 23 lncRNAs (**Supplementary Table 18**). Based on the above results, the corresponding regulatory network was constructed as shown in **Figure 6A**. In this network, MCL1 showed a wider range of regulatory effects, while the scope of TGFB1 was relatively limited. The two might synergistically participate in related biological processes or disease progression through different levels of regulation. Further, an interaction network between genes potentially related to biomarkers was successfully constructed (**Figure 6B**). The results showed that there were 20 human-related genes interacting with biomarkers, and all of them were significantly related to functions such as positive regulation of mitochondrion organization, regulation of intrinsic apoptotic signaling pathway, regulation of mitochondrion organization, intrinsic apoptotic signaling pathway, regulation of apoptotic signaling pathway, apoptotic mitochondrial changes, and cellular response to transforming growth factor beta stimulus. These findings provided important clues for in-depth analysis of the functional mechanism of biomarkers in organisms.

Finally, through DSigDB database prediction, it was found that there were 331 and 132 potential drugs that might interact with MCL1 and TGFB1, respectively (**Supplementary Table 19**). The study further selected the top 20 drugs according to the screening criteria and constructed the drug-biomarker relationship network diagram based on this (**Figure 6C**). In this network diagram, biomarkers could interact with compounds such as curcumin, ellagic acid, aspirin, domperidone, and acetaldehyde, which provided a potential idea of “multi-target synergistic intervention” for disease treatment. In addition, the top 1 drug screened was domperidone, which was used for subsequent molecular docking. The molecular docking results showed that the docking scores of the 2 biomarkers with domperidone were both lower than -5.0 kcal/mol, indicating good binding activity. Specifically, for MCL1, its Vina score with domperidone was -7.7 kcal/mol, and during molecular docking, interactions with bond lengths of 3.1 Å, 2.2 Å, and 3.1 Å were formed, and the corresponding binding sites were ARG-88, GLN-89, and ARG-215; while for TGFB1, its Vina score with domperidone was -9.3 kcal/mol, and during molecular docking, interactions were formed at sites such as PHE-60, VAL-61, SER-33, VAL-34, and THR-74 with bond lengths of 2.0 Å, 3.2 Å, 2.9 Å, 2.9 Å, and 3.4 Å, respectively. The 3D docking diagrams provide a detailed spatial view of the binding interfaces between domperidone and the identified biomarkers. Domperidone exhibited strong affinity for the binding pockets of both proteins, characterized by stable hydrogen bonding networks. The precise H-bond distances, ranging from 2.0 Å to 3.4 Å, are explicitly illustrated in the 3D models, confirming the potent and specific interactions between the compound and the biomarkers (MCL1 and TGFB1) (**Table 1**; **Figure 6D**).

**Table 1:** Molecular docking results.

Gene	Molecule	Vina score(kcal/mol)
MCL1	domperidone	-7.7
TGFB1	domperidone	-9.3

### 3.5 Validation of the identified biomarkers by RT-qPCR and western blot

**Figure 7:**
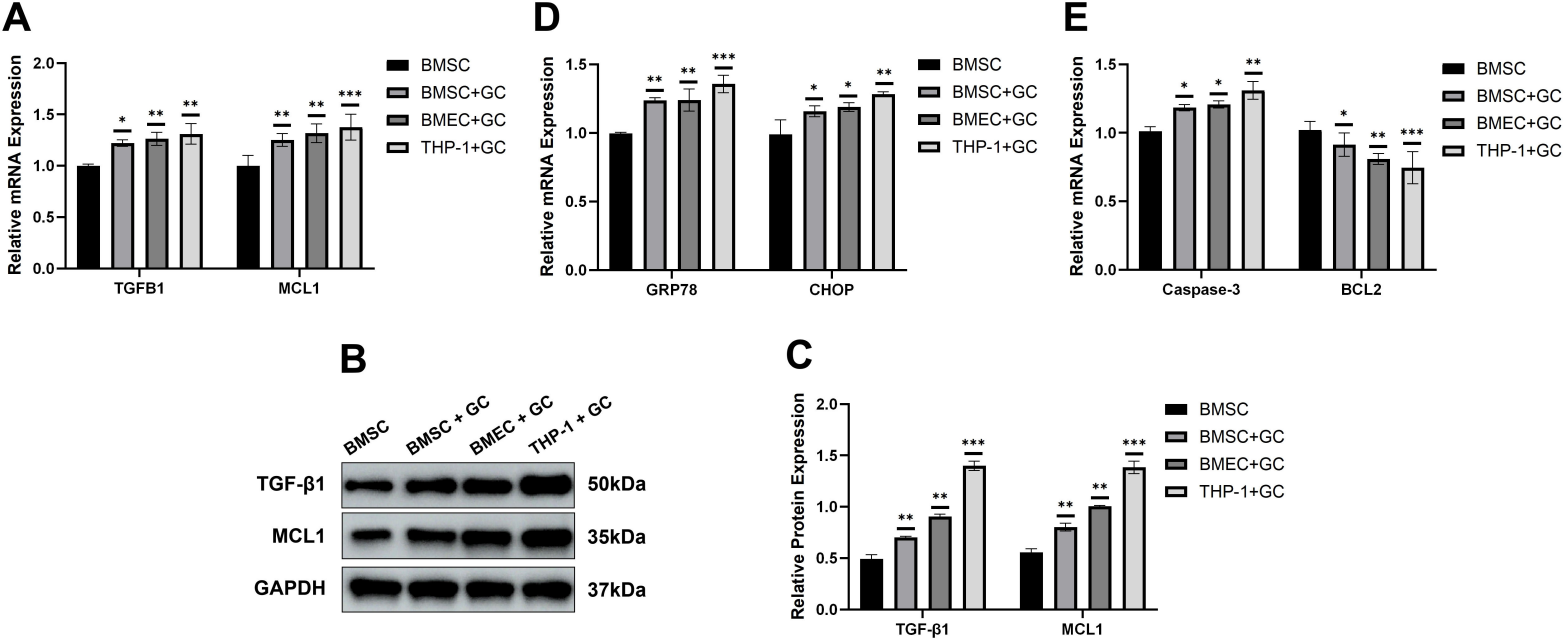
Validation of candidate genes by RT-qPCR and Western blot. **(A)** mRNA expression levels of the two biomarkers TGFB1 and MCL1 selected by bioinformatics; **(B)** Representative Western blot bands of the two biomarkers TGFB1 and MCL1 selected by bioinformatics; **(C)** Quantification of TGFB1 and MCL1 protein expression; **(D)** mRNA expression levels of the ERS biomarkers markers GRP78 and CHOP; **(E)** mRNA expression levels of the RCD biomarkers markers Caspase-3 and BCL2. *p < 0.05, **p < 0.01 and ***p < 0.001 compared with the BMSC group; ERS, endoplasmic reticulum stress; RCD, Regulated Cell Death.

By quantitatively detecting the mRNA and protein expression levels of TGFB1 and MCL1 in different cells (BMSC, BMEC, THP-1) after GC treatment, the differences in the expression levels of these two biomarkers selected by bioinformatics were verified at the molecular experimental level. Specifically, RT-qPCR results showed that both genes were significantly upregulated after GC treatment in the above cell models that can simulate the pathological environment of ONFH (**Figure 7A**). Notably, Western blot analysis of TGFB1 and MCL1 proteins revealed a consistent expression trend (**Figures 7D, E**). In addition, RT-qPCR analysis demonstrated that after GC treatment, the expression of endoplasmic reticulum stress (ERS) markers, including GRP78 and CHOP, as well as the pro-apoptotic gene Caspase-3, was markedly increased in BMSC, BMEC, and THP-1 cells. In contrast, the expression of the anti-apoptotic gene BCL2 was decreased (**Figures 7B, C**). These findings indicate that the ONFH-like pathological environment induced by GC treatment successfully activated the ERS–RCD axis, which is consistent with the bioinformatics observation that TGFB1 and MCL1 were both significantly upregulated in clinical samples of the ONFH group. This experimental–computational cross-validation provides direct evidence supporting the reliability of the biomarkers identified through bioinformatics. Furthermore, the differential expression of TGFB1 and MCL1 among different cell types under GC treatment provides additional insights into their cell-type–specific expression patterns and offers experimental evidence at the cellular level for understanding their potential roles in the pathogenesis and progression of ONFH.

## 4 Discussion

Previous studies indicate that endoplasmic reticulum stress (ERS) is abnormally activated in ONFH, driving cellular damage via inflammation, oxidative stress, and lipid metabolism disorders, while regulated cell death (RCD)—including apoptosis, autophagy, pyroptosis, and ferroptosis—directly governs osteocyte loss and femoral head collapse; together they promote ONFH progression, though their crosstalk remains poorly understood (33, 34). It should be acknowledged that the training dataset compared SONFH with non-SONFH patients rather than healthy individuals. Consequently, the identified biomarkers (TGFB1 and MCL1) primarily reflect the molecular distinctions between these two clinical subtypes. However, the subsequent validation in the test set (GSE74089), which utilized cartilage tissue from healthy controls, as well as the consistent upregulation observed in glucocorticoid-treated cell models, demonstrates that these markers are not only subtype-specific but also significantly dysregulated compared to normal physiological states. This cross-validation across different control types and biological sources reinforces the robustness of TGFB1 and MCL1 as key pathological mediators in SONFH. In this study, we identified 16 hub genes through the PPI network and four algorithms, and further confirmed TGFB1 and MCL1 as biomarkers using machine learning and Wilcoxon testing, both significantly upregulated in ONFH (p < 0.05). ROC analysis showed strong diagnostic performance (AUC > 0.85), and a nomogram model achieved high predictive accuracy (AUC = 0.897, Hosmer–Lemeshow p = 0.171) with net clinical benefit above zero. The diagnostic efficacy suggested by the ROC and nomogram analyses is reinforced by the biological evidence observed in the experimental validation. The significant upregulation of TGFB1 and MCL1 in glucocorticoid-induced cell models provides a direct biological basis for their inclusion in the predictive model. The integration of these markers into a scoring system reflects their potential as biological surrogates for the cellular stress and regulated cell death processes occurring in the early stages of SONFH. Enrichment, immune infiltration, drug prediction, molecular docking, and experimental validation suggested their involvement in pathogen-related pathways, correlation with plasmacytoid dendritic cells, and strong binding to domperidone (Vina score ≤ –7.7 kcal/mol). Experimental validation in glucocorticoid-treated cell models confirmed the upregulation of TGFB1 and MCL1 in ONFH, consistent with clinical samples, supporting the reliability of these bioinformatically-identified biomarkers.

A significant finding of this study is the consistent upregulation of TGFB1 and MCL1 across substantially different biological compartments. While the serum-derived training data reflect systemic molecular alterations, the validation in cartilage tissue confirms that these changes are intrinsically linked to the localized necrotic process in the femoral head. The coexistence of these biomarkers in both systemic circulation and the target lesion suggests that local ERS and RCD processes may release molecular signals into the peripheral blood, or that ONFH involves a systemic predisposition that manifests locally. This cross-validation between liquid biopsy (serum) and solid tissue (cartilage) significantly enhances the reliability and clinical utility of TGFB1 and MCL1, demonstrating their potential for both non-invasive early diagnosis and as therapeutic targets within the local pathological microenvironment.

This study identified TGFB1 as one of the biomarkers associated with ONFH through bioinformatics, and this finding is supported by existing clinical studies. Li et al. (35), through the study of femoral head specimens from patients with non-traumatic osteonecrosis of the femoral head, found that TGF-β1 expression was significantly decreased in non-traumatic osteonecrosis of the femoral head tissue (P<0.01), and was closely related to the imbalance of CD4+/CD8+ T cell ratio. This study further indicated that the decreased expression of TGF-β1 was associated with impaired femoral head repair capacity and abnormal immune function, suggesting that TGF-β1 may be a key regulatory factor in the pathogenesis of ONFH.

GSEA analysis suggested that TGFB1 may participate in regulating both the enteropathogenic Escherichia coli (EAE) TIR–actin signaling and the Toll-like receptor (TLR) 1/2/4–NF-κB pathways. Notably, the TLR–NF-κB axis has been repeatedly implicated in the development of osteonecrosis of the femoral head (ONFH). As reported by Okura et al (36), activation of TLR7 and TLR9 is required for the onset of non-traumatic ONFH, and exposure to glucocorticoids can further exacerbate disease progression by suppressing NF-κB signaling. Subsequent studies have also highlighted the pivotal role of the TLR4/NF-κB pathway in glucocorticoid-induced ONFH. Moreover, treatment with the natural compound calycosin was shown to mitigate ONFH-related pathological changes by downregulating this signaling cascade (37). This suggests that TGFB1 may regulate the host response to pathogen stimulation through multiple signaling pathways, providing direction for further understanding its role in inflammation-related pathological processes of ONFH. GSVA further revealed the effect of TGFB1 expression on pathway activity, with 15 pathways activated and 2 suppressed in the high-expression group. Among the enriched pathways, two stand out for their biological relevance. The inflammatory response and PI3K–AKT–mTOR signaling cascades were both markedly activated (t > 2, p < 0.05). The first is likely to amplify local inflammatory injury within ONFH lesions (38, 39), whereas the second—an essential hub for cellular metabolism and survival—appears to promote lesion persistence by reshaping osteocyte energy metabolism and maintaining aberrant survival signaling (40, 41). In contrast, pathways associated with pancreatic β-cell function and heme metabolism were significantly suppressed (t < –2, p < 0.05). Such systemic metabolic disturbances could in turn alter the bone microenvironment and indirectly accelerate disease progression (1, 42, 43). While the enrichment analysis primarily revealed immune signaling pathways, this is consistent with the systemic nature of serum-derived transcriptomic data. In ONFH, particularly steroid-induced cases, immune dysregulation is not merely a bystander but a critical mediator of bone pathology (38). For instance, TGFB1 is a pleiotropic cytokine that orchestrates both immune responses and bone remodeling. The enrichment of TLR and NF-κB pathways suggests that chronic systemic inflammation may trigger endoplasmic reticulum stress (ERS) in bone cells, eventually culminating in RCD (37). Consequently, the identified biomarkers, TGFB1 and MCL1, likely capture the systemic inflammatory state that predisposes or contributes to the localized necrotic process in the femoral head by bridging immune activation and cellular stress responses. Taken together, these observations suggest that TGFB1 serves as a multifaceted regulator in ONFH, coordinating diverse signaling activities that collectively shape disease onset and progression.

This study identified 20 human genes closely related to TGFB1, which are mainly involved in “response to transforming growth factor beta stimulus,” “regulation of apoptotic signaling pathway,” and “mitochondrial organization.” Studies have found that methylprednisolone significantly inhibits angiogenesis of endothelial progenitor cells by inducing apoptosis, disrupting normal mitochondrial structure, and interfering with mitochondrial function. This study confirmed that glucocorticoids can induce mitochondria-mediated apoptosis and inhibit endothelial progenitor cell angiogenesis, and the PTEN inhibitor VO-OHpic can significantly improve osteonecrosis of the femoral head by activating the Nrf2 signaling pathway and inhibiting the mitochondrial apoptosis pathway (44). These findings highlight the central role of TGFB1 in cell signaling and apoptosis regulation, and suggest that it may affect osteocyte survival by influencing mitochondrial function, thereby promoting the development of ONFH. Moreover, at the genomic and cellular levels, TGFB1 maps to chromosome 19 and exhibits predominant cytoplasmic expression. This distribution allows the protein to promptly sense external cues and modulate downstream signaling activity, forming the spatial basis for its participation in both the activation and suppression of signaling pathways during the pathological progression of ONFH.

Myeloid cell leukemia-1 (MCL1) is an anti-apoptotic member of the Bcl-2 family that acts through the mitochondrial pathway to preserve cell survival. Beyond its role in apoptosis inhibition, MCL1 contributes to the regulation of the cell cycle, supports immune cell function, and helps maintain tissue homeostasis. Aberrant expression or loss of MCL1 function has been implicated in defective cell survival and in the pathogenesis of multiple diseases (45). Consistent with its multifunctional roles, GSEA analysis indicated that MCL1 participates in several key signaling cascades, including the epidermal growth factor (EGF)/EGFR/ACTIN and interferon (IFN)/RIPK1/3 pathways. The EGF–EGFR–ACTIN axis is primarily associated with cell proliferation and cytoskeletal remodeling (46), whereas the IFN–RIPK1/3 pathway contributes to immune regulation and cell death control (47). The enrichment of these pathways suggests MCL1 as a potential integrator of hypoxia, inflammatory, and apoptotic signaling during ONFH progression. Such convergence may place MCL1 at a pivotal point where metabolic stress, inflammation, and cell survival intersect. This mechanistic implication provides a plausible explanation for how altered MCL1 expression contributes to osteocyte vulnerability and tissue damage under ischemic conditions (48, 49). In contrast, KRAS signaling (DN), spermatogenesis, and heme metabolism pathways were markedly suppressed (t < –2, p < 0.05), which may impair normal cellular proliferation and disturb metabolic homeostasis. Such alterations could weaken bone repair capacity and establish a vicious cycle of *“worsening injury and inadequate regeneration” (*50). At the genomic and subcellular levels, MCL1 is positioned on chromosome 1 and predominantly localized in the cytoplasm. Within the 1p36 region, the DR3/Apo3 death receptor engages adaptor molecules TRADD and FADD to trigger downstream apoptotic signaling (51), underscoring the importance of chromosome 1–encoded genes in apoptosis regulation. Cytoplasmic anti-apoptotic mechanisms also converge at the Akt–Bad–Bcl-2 axis: once Akt phosphorylates and inactivates Bad, it subsequently promotes the expression of Bcl-2 family proteins, thereby sustaining cell survival (9). Under glucocorticoid-induced endoplasmic reticulum (ER) stress, activation of the PERK/CHOP pathway disrupts the stability of cytoplasmic anti-apoptotic proteins. When ER stress becomes prolonged, mitochondrial outer membrane permeability is altered, resulting in cytochrome c release and caspase-3 activation, ultimately driving osteocyte apoptosis (33). Collectively, these findings suggest that MCL1, located on chromosome 1 and primarily expressed in the cytoplasm, may serve a vital cytoprotective function in the pathogenesis of glucocorticoid-induced osteonecrosis of the femoral head (ONFH) by modulating key checkpoints within the ER stress–mitochondrial apoptosis axis.

The imbalance in cellular composition revealed by immune infiltration analysis mirrors a key pathological feature of the ONFH microenvironment. An excessive accumulation of myeloid-derived suppressor cells (MDSCs)—known for their immunosuppressive properties—within ONFH lesions may impair the efficient clearance of necrotic bone tissue and disturb the fragile equilibrium between inflammation and repair. Such dysregulation likely contributes to persistent inflammatory activity and defective healing responses (52, 53). In contrast, CD56^bright^ natural killer (NK) cells were markedly enriched in the control group. These cells exhibit a dual role: they exert cytotoxic activity against abnormal targets and secrete cytokines that promote tissue repair, thereby helping to preserve immune homeostasis and protect bone integrity (54). During the development of steroid-induced osteonecrosis of the femoral head, dendritic cells form intricate communication networks with T cells via the RANK–RANKL signaling axis. Through this interaction, they fine-tune Th1-driven inflammatory activation and simultaneously regulate osteoclast function, ultimately influencing bone resorption dynamics (38). The functional imbalance of these immune cell populations not only underscores the immunological foundation of ONFH pathogenesis but also offers valuable insight into its underlying molecular mechanisms. Understanding these alterations may further aid in identifying novel therapeutic targets and developing immunomodulatory strategies for ONFH management. In terms of cell–cell associations, most DICs showed significant positive correlations, with the strongest observed between Th1 cells and MDSCs (cor = 0.88, p < 0.001), suggesting a runaway regulatory loop that forms an “immunosuppressive trap.” The only negative correlation was between immature B cells and CD56bright NK cells (cor = –0.32, p < 0.05), reflecting a breakdown in the balance between innate and adaptive immunity. At the biomarker level, both MCL1 and TGFB1 showed the strongest positive correlation with plasmacytoid dendritic cells (pDCs) (cor = 0.87 and 0.81, respectively; p < 0.05). MCL1 may promote abnormal immune regulation by sustaining pDC survival (55), while TGFB1 may drive a vicious “anti-inflammatory–tolerance” cycle with pDCs (56), together reinforcing the immunosuppressive microenvironment. These findings point to a potential “biomarker–DIC–immune microenvironment” axis as a core pathway in the pathogenesis of ONFH. The theoretical and clinical value of this study lies in contributing to the understanding of the ONFH–ERS/RCD immune microenvironment. The clear association between biomarkers and DICs provides potential therapeutic targets, such as MCL1 inhibitors (55) and TGFB1-neutralizing antibodies. Differences in the proportions of MDSCs and CD56bright NK cells may aid in early diagnosis, while also expanding therapeutic strategies aimed at modulating the immune microenvironment. The experimental observation that ERS and RCD markers were co-expressed with TGFB1 and MCL1 provides direct evidence for the involvement of the ERS-RCD axis in ONFH. The simultaneous elevation of CHOP, a key mediator of ERS-induced apoptosis, and the identified biomarkers suggests a potential regulatory network where TGFB1 and MCL1 may modulate or respond to cellular stress signals. This synchrony validates the initial hypothesis that the identified biomarkers are intrinsically linked to the programmed cell death pathways driven by endoplasmic reticulum dysfunction.

This study employed the DSigDB database to predict potential compounds targeting the biomarkers MCL1 and TGFB1. After applying the predefined screening criteria, the top 20 candidate molecules were selected to establish a drug–biomarker interaction network. Network analysis highlighted several compounds—including curcumin, ellagic acid, aspirin, domperidone, and acetaldehyde—that were predicted to interact with both MCL1 and TGFB1, suggesting possible shared therapeutic relevance.

As the top-ranked candidate, domperidone exhibited molecular docking results that further supported its potential as a targeted therapeutic agent for ONFH. It showed a binding affinity of –7.7 kcal/mol with MCL1 and –9.3 kcal/mol with TGFB1, both surpassing the conventional threshold of –5.0 kcal/mol. These values indicate a strong and stable interaction between domperidone and the two biomarkers, providing a plausible molecular basis for targeted modulation in ONFH.

Domperidone, a dopamine receptor antagonist, is primarily prescribed for nausea, vomiting, and gastrointestinal motility disorders, and is also known for its lactogenic properties (57). Although no clinical studies have directly examined its effects on bone metabolism, accumulating evidence indicates that domperidone stimulates pituitary prolactin secretion, thereby increasing serum prolactin levels—a change that may indirectly influence bone health. Hyperprolactinemia is known to affect bone metabolism through two main mechanisms. First, it suppresses the hypothalamic–pituitary–gonadal axis, leading to reduced estrogen and testosterone levels and, consequently, secondary osteoporosis. Second, prolactin exerts direct effects on osteoblasts and osteoclasts, disturbing the balance of bone remodeling (58). In this context, the present molecular docking analysis demonstrated that domperidone binds strongly to the ONFH-related proteins MCL1 and TGFB1, suggesting a potential direct targeting effect in bone tissue. Taken together, these findings imply that domperidone may exert a dual mode of action in bone health—indirectly via prolactin-mediated endocrine regulation and directly through molecular interactions with key proteins involved in osteonecrosis. Nevertheless, the clinical implementation of domperidone for ONFH therapy necessitates a rigorous evaluation of the benefit-risk ratio. A primary concern is domperidone-induced hyperprolactinemia, arising from its role as a dopamine receptor antagonist. Elevated serum prolactin levels can suppress the hypothalamic-pituitary-gonadal axis, leading to diminished estrogen or androgen levels, which may impair systemic bone metabolism and decrease bone mineral density (59). Therefore, while domperidone potentially offers localized benefits by interacting with the TGFB1/MCL1 axis, its systemic adverse effects on bone homeostasis must be effectively mitigated. It is proposed that domperidone-based interventions should be integrated into a comprehensive anti-osteoporotic framework. Specifically, the co-administration of bone-protective agents, such as bisphosphonates or denosumab, alongside the routine monitoring of serum prolactin, could provide a more balanced therapeutic strategy, ensuring the preservation of bone structural integrity while addressing the necrotic lesions (59, 60).

This discovery carries dual significance. On one hand, it points to the possibility of repurposing clinically available drugs such as aspirin, and of harnessing natural bioactive compounds like curcumin for the treatment of ONFH. On the other, the identification of shared interactions between multiple compounds and both MCL1 and TGFB1 suggests a rationale for multi-target synergistic therapy. By combining agents that simultaneously modulate inflammatory and cell survival–related pathways, it may be possible to achieve a broader and more balanced therapeutic effect in ONFH. Such a concept resonates with the emerging paradigm in modern pharmacology that favors multi-target intervention over single-molecule strategies.

However, several limitations of this study should be acknowledged. First, the use of peripheral blood samples for the training set may bias the bioinformatics findings toward systemic immune activation, which may not fully reflect the localized mechanical and metabolic alterations occurring within the bone microenvironment. Second, although TGFB1 and MCL1 expression was validated at both the transcriptional and protein levels, additional functional experiments—such as mitochondrial assays (e.g., JC-1 staining and ATP measurements)—were not included in the present study. These experiments may provide further mechanistic insights into ER stress–related regulated cell death and will be explored in future investigations. Third, the sample sizes for both the predictive modeling and external validation cohorts were relatively small. The external validation dataset (GSE74089) included only four samples per group, which may limit generalizability and increase the risk of Type II errors. Therefore, the nomogram and ROC analyses based on this cohort should be regarded as proof-of-concept evidence rather than definitive clinical tools. Although *post-hoc* power analysis showed that the statistical power for TGFB1 and MCL1 exceeded 0.8, validation in broader clinical populations is still required. Future studies with larger, multi-center, and prospectively collected cohorts are needed to confirm the diagnostic value of these biomarkers in SONFH.

In summary, this study uncovered a potential role of the endoplasmic reticulum stress–regulated cell death (ERS–RCD) axis in the development of osteonecrosis of the femoral head (ONFH). TGFB1 and MCL1 were identified as key biomarkers associated with inflammatory and survival pathways, while domperidone emerged as a potential therapeutic candidate with multi-target activity. These findings deepen understanding of ONFH pathogenesis and suggest new directions for targeted and repurposed therapies, though further experimental and clinical validation is still needed. However, its clinical utilization remains contingent upon the development of integrated strategies that manage prolactin-related skeletal risks through combined anti-osteoporotic therapies.

## Data Availability

The datasets analyzed for this study can be found in the Gene Expression Omnibus (GEO) repository (https://www.ncbi.nlm.nih.gov/geo/) under accession numbers GSE123568 and GSE74089.

## Glossary

ERS: Endoplasmic Reticulum Stress
RCD: Regulated Cell Death
ONFH: Osteonecrosis of the Femoral Head
DEGs: Differentially Expressed Genes
PPI: Protein-Protein Interaction
ROC: Receiver Operating Characteristic
AUC: Area Under the ROC Curve
GC: Glucocorticoid
BMSC: Bone Marrow Mesenchymal Stem Cells
BMEC: Brain Microvascular Endothelial Cells
RT-qPCR: Reverse Transcription-Quantitative Polymerase Chain Reaction
GEO: Gene Expression Omnibus
GPL: Gene Expression Platform (affymetrix platform identifier, e.g., GPL15207, GPL13497)
GSEA: Gene Set Enrichment Analysis
KEGG: Kyoto Encyclopedia of Genes and Genomes
GO: Gene Ontology
DO: Disease Ontology
DO-KB: The Disease Ontology Knowledgebase
STRING: Search Tool for the Retrieval of Interacting Genes/Proteins
DMNC: Density of Maximum Neighborhood Component
MCC: Maximum Clique Centrality
MNC: Maximum Neighborhood Component
LASSO: Least Absolute Shrinkage and Selection Operator
SVM-RFE: Support Vector Machine-Recursive Feature Elimination
XGBoost: eXtreme Gradient Boosting
TF: Transcription Factor
miRNA: MicroRNA
lncRNA: Long Non-Coding RNA
ENCORI: Encyclopedia of RNA Interactomes
DSigDB: Drug Signatures Database
Uniprot: Universal Protein Resource
CB-DOCK2: Computer-Aided Drug Design Dock 2
FBS: Fetal Bovine Serum
PMA: Phorbol 12-Myristate 13-Acetate
PBS: Phosphate-Buffered Saline
GAPDH: Glyceraldehyde-3-Phosphate Dehydrogenase
NOD: Nucleotide-Binding Oligomerization Domain
NF-κB: Nuclear Factor Kappa-Light-Chain-Enhancer of Activated B Cells
IL-6: Interleukin-6
JAK2: Janus Kinase 2
STAT3: Signal Transducer and Activator of Transcription 3
mTOR: Mechanistic Target of Rapamycin
MDSCs: Myeloid-Derived Suppressor Cells
NK: Natural Killer
pDCs: Plasmacytoid Dendritic Cells
EGF: Epidermal Growth Factor
EGFR: Epidermal Growth Factor Receptor
IFN: Interferon
RIPK1/3: Receptor-Interacting Serine/Threonine-Protein Kinase 1/3
DR3/Apo3: Death Receptor 3/Apo-3
TRADD: Tumor Necrosis Factor Receptor Type 1-Associated Death Domain Protein
FADD: Fas-Associated Death Domain Protein
PERK: Protein Kinase R-Like Endoplasmic Reticulum Kinase
CHOP: C/EBP Homologous Protein
Akt: Protein Kinase B
Bcl-2: B-Cell Lymphoma 2
TLR: Toll-Like Receptor
PTEN: Phosphatase and Tensin Homolog
Nrf2: Nuclear Factor Erythroid 2-Related Factor 2
MCL1: Myeloid Cell Leukemia-1
MSigDB: Molecular Signatures Database
GSVA: Gene Set Variation Analysis
ssGSEA: Single-Sample Gene Set Enrichment Analysis
AUC: Area Under the Curve (consistent with ROC-AUC, used in nomogram evaluation)
CDF: Cumulative Distribution Function
Z-score: Standard Score (used in immune cell infiltration normalization)
