# Supplementary figures and images for: Exploration of potential mechanisms and biomarkers related to ERS-associated RCD in steroid-induced osteonecrosis of the femoral head based on bioinformatics, with experimental validation
Source: Front Endocrinol (Lausanne). 2026 May 18;17:1734283. doi: 10.3389/fendo.2026.1734283 (PMC13223048; doi:10.3389/fendo.2026.1734283)

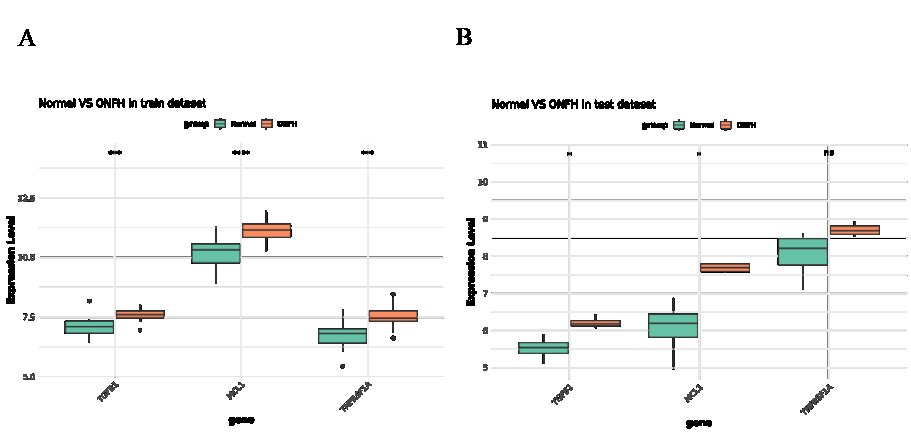

Supplement: Supplementary file 16 [file Image1.jpeg]
