# Supplementary material for: Exploration of potential mechanisms and biomarkers related to ERS-associated RCD in steroid-induced osteonecrosis of the femoral head based on bioinformatics, with experimental validation
Source: Front Endocrinol (Lausanne). 2026 May 18;17:1734283. doi: 10.3389/fendo.2026.1734283 (PMC13223048; doi:10.3389/fendo.2026.1734283)
Supplement: Supplementary file 20 [file Table4.docx]

Supplementary Material

# Supplementary Tables

Supplementary Table 1. Acquisition of ERGs.

Supplementary Table 2. Acquisition of RRGs.

Supplementary Table 3. Acquisition of DEGs.

Supplementary Table 4. Acquisition of candidate genes.

Supplementary Table 5. Result of GO analysis.

Supplementary Table 6. Result of KEGG analysis

Supplementary Table 7. Result of DO enrichment analysis.

Supplementary Tables 8-11. Gene ranking results based on four algorithms (Degree, DMNC, MCC, MNC).

Supplementary Table 12. Acquisition of core genes.

Supplementary Table 13. GSEA of gene MCL1.

Supplementary Table 14. GSEA of gene TGFB1.

Supplementary Table 15. GSVA of gene MCL1.

Supplementary Table 16. GSVA of gene TGFB1.

Supplementary Table 17. Correlation analysis between biomarkers and DICs.

Supplementary Table 18. Regulatory miRNAs prediction results of biomarkers.

Supplementary Table 19. Result of drug prediction (for biomarkers).

Supplementary Table S1. Related primer sequences.
