# Supplementary material for: Exploration of potential mechanisms and biomarkers related to ERS-associated RCD in steroid-induced osteonecrosis of the femoral head based on bioinformatics, with experimental validation
Source: Front Endocrinol (Lausanne). 2026 May 18;17:1734283. doi: 10.3389/fendo.2026.1734283 (PMC13223048; doi:10.3389/fendo.2026.1734283)
Supplement: Supplementary file 21 [file Table5.docx]

**Supplementary Table S1. Related primer sequences.**

| **Primer** | **Sequence (5'-3')** | |
| --- | --- | --- |
| TGFB1 F | TCAACGCAGGGTTCACTACC | |
| TGFB1 R | GAAGTTGGCATGGTAGCCCT | |
| MCL1 F | CGGCAGTCGCTGGAGATTAT |  |
| MCL1 R | TCCACAAACCCATCCTTGGAA |  |
| GRP78 F | GGAGGAGGACAAGAAGGAGG |  |
| GRP78 R | GTGGTGATGTTCTTCTGGGT |  |
| CHOP F | CAGAACCAGCAGAGGTCACA |  |
| CHOP R | AGCTGTGCCACTTTCCTTTC |  |
| Caspase-3 F | GAAATTGTGGAATTGATGCGTG |  |
| Caspase-3 R | CTACAACGATCCCCTCTGAAAAA |  |
| BCL2 F | GGTGGGGTCATGTGTGTGG |  |
| BCL2 R | CGGTTCAGGTACTCAGTCATCC |  |
| Internal reference H-GAPDH F | ATGGGCAGCCGTTAGGAAAG |  |
| Internal reference H-GAPDH R | AGGAAAAGCATCACCCGGAG |  |
